# Living Arrangements, Family Support, and Health Outcomes Among Older Adults in China

**DOI:** 10.1093/geroni/igaf122.3302

**Published:** 2025-12-31

**Authors:** Sijiu Wang, Rui Wu

**Affiliations:** Tsinghua University, Beijing, Beijing, China (People’s Republic); Tsinghua University, Beijing, Beijing, China (People’s Republic)

## Abstract

As demographic shifts and urbanization reshape family structures in China, understanding the relationship between living arrangements and health outcomes among older adults is critical. This study examines how different living arrangements influence self-rated health, mental health, and functional status, and whether these effects are mediated by family support. Using three waves (2013, 2015, and 2018) of the China Health and Retirement Longitudinal Study (CHARLS), we categorized living arrangements into four groups: living alone without children in the same city, living alone with children nearby, living with a spouse only, and living with others beyond a spouse. Lagged models addressed potential reverse causality, while mediation analyses explored three key support mechanisms: informal care, frequency of child contact (in-person and technology-based), and financial support. Findings suggest that living alone with children nearby and living with a spouse are associated with better self-rated and mental health compared to living alone without children in the same city. Living with others beyond a spouse is linked to improved mental health. Mediation analyses reveal that informal care receipt supports functional health, while all forms of regular child contact enhance mental and functional health. Financial support is associated with improvements across all health outcomes. These results underscore the significance of both living arrangements and family support in shaping older adults’ health. Policymakers should consider strategies to promote intergenerational proximity and alternative support mechanisms for aging populations.

